# Evidence of Naturalized Stress-Tolerant Strains of Escherichia coli in Municipal Wastewater Treatment Plants

**DOI:** 10.1128/AEM.00143-16

**Published:** 2016-08-30

**Authors:** Shuai Zhi, Graham Banting, Qiaozhi Li, Thomas A. Edge, Edward Topp, Mykola Sokurenko, Candis Scott, Shannon Braithwaite, Norma J. Ruecker, Yutaka Yasui, Tim McAllister, Linda Chui, Norman F. Neumann

**Affiliations:** a School of Public Health, University of Alberta, Edmonton, Alberta, Canada; b Environment Canada, Burlington, Ontario, Canada; c Agriculture and Agri-Food Canada, London, Ontario, Canada; d Water Quality Services, Calgary, Alberta, Canada; e Agriculture and Agri-Food Canada, Lethbridge, Alberta, Canada; f Department of Laboratory Medicine and Pathology, University of Alberta, Edmonton, Alberta, Canada; g Environmental Microbiology Program, Provincial Laboratory for Public Health, Edmonton, Alberta, Canada; Wageningen University

## Abstract

Escherichia coli has been proposed to have two habitats—the intestines of mammals/birds and the nonhost environment. Our goal was to assess whether certain strains of E. coli have evolved toward adaptation and survival in wastewater. Raw sewage samples from different treatment plants were subjected to chlorine stress, and ∼59% of the surviving E. coli strains were found to contain a genetic insertion element (IS*30*) located within the *uspC-flhDC* intergenic region. The positional location of the IS*30* element was not observed across a library of 845 E. coli isolates collected from various animal hosts or within GenBank or whole-genome reference databases for human and animal E. coli isolates (*n* = 1,177). Phylogenetics clustered the IS*30* element-containing wastewater E. coli isolates into a distinct clade, and biomarker analysis revealed that these wastewater isolates contained a single nucleotide polymorphism (SNP) biomarker pattern that was specific for wastewater. These isolates belonged to phylogroup A, possessed generalized stress response (RpoS) activity, and carried the locus of heat resistance, features likely relevant to nonhost environmental survival. Isolates were screened for 28 virulence genes but carried only the *fimH* marker. Our data suggest that wastewater contains a naturalized resident population of E. coli. We developed an endpoint PCR targeting the IS*30* element within the *uspC-flhDC* intergenic region, and all raw sewage samples (*n* = 21) were positive for this marker. Conversely, the prevalence of this marker in E. coli-positive surface and groundwater samples was low (≤5%). This simple PCR assay may represent a convenient microbial source-tracking tool for identification of water samples affected by municipal wastewater.

**IMPORTANCE** The results of this study demonstrate that some strains of E. coli appear to have evolved to become naturalized populations in the wastewater environment and possess a number of stress-related genetic elements likely important for survival in this nonhost environment. The presence of non-host-adapted strains in wastewater challenges our understanding of using E. coli as a microbial indicator of wastewater treatment performance, suggesting that the E. coli strains present in human and animal feces may be very different from those found in treated wastewater.

## INTRODUCTION

Escherichia coli, a common Gram-negative facultative anaerobe, has been one of the most-studied microorganisms since its discovery in 1885 (1). It has been proposed to have two principal habitats: the intestinal tracts of mammals and birds and the nonhost environment (water/sediment). Survival and colonization in these distinct habitats are mediated by adaptations to nutrient availability, temperature, pH, osmolality, solar radiation, and the presence of competitive microflora. Although E. coli has been used as a water quality indicator of fecal contamination for years, several studies have found that E. coli can grow outside the gastrointestinal tracts of its animal hosts in tropical and subtropical environments (2–4). Naturalized E. coli strains have also been described for sand, sediments, and water from temperate climates (5–9). The presence of E. coli in these environments indicates that some E. coli strains may survive and replicate outside animal host reservoirs.

For bacteria, survival and adaptation in different environments are necessary for their continued evolutionary success, ultimately leading to genetic differences between populations. These differences may arise through mutations in regulatory sequences (10), acquisition of functional genes through transduction or horizontal gene transfer (11, 12), random point mutations (13), homologous recombination (14), and/or conjugation (15). Mutations that confer a fitness advantage and enhance survival in certain environments can lead to natural selection of subpopulations. For example, in a long-term *in vitro* evolutionary experiment on E. coli populations, it was observed that under conditions of citrate abundance and glucose limitation, certain E. coli strains acquired an aerobically expressed promoter for the expression of a previously silent citrate transporter that enabled these strains to use citrate as an energy source (10).

The intestinal tracts of different animal hosts also represent unique environments for bacteria. Host-specific bacterial strains have been found in ducks, chickens, humans, pigs, and horses (16, 17). For example, a human-specific E. coli clone of the B2 lineage was reported by Clermont et al. (18), and its presence was consistent across Europe, Africa, and South America. In addition, when wild Bartonella strains were inoculated into different rodent species, infection was found in rats only if the inoculated strains were isolated from the same rodent species or phylogenetically close species (19). Recently, we identified single nucleotide polymorphic (SNP) biomarkers of host specificity in E. coli isolates from 15 different human and animal species by using logic regression modeling of intergenic DNA sequences (20). In preliminary experiments evaluating these host-specific biomarkers of E. coli isolates from humans and animals, we observed that E. coli isolates obtained from wastewater appeared to be genetically different from human and animal isolates.

Municipal wastewater represents a very different environment for E. coli compared to the gastrointestinal tract. Wastewater has a very unique microbial composition (21), and its chemical composition encompasses a wide range of organic (detergents, antibiotics, and personal care products) and inorganic (i.e., nutrients [nitrogen and phosphorus] and metals [mercury, lead, and copper]) compounds (22). Consequently, we hypothesized that the evolutionary selection forces imposed by wastewater treatment may drive certain E. coli strains to adopt survival strategies in the wastewater environment (e.g., treatment resistance), potentially leading to the evolution of naturalized populations of E. coli. The objective of this study was to assess whether certain strains of E. coli in wastewater are genetically distinct from fecal populations of this bacterium.

## MATERIALS AND METHODS

### E. coli isolates.

In total, 1,426 E. coli strains were used in this study (Table 1). Among them, 845 E. coli strains were collected from 15 different animal host species, including humans. Seventy E. coli strains were isolated from chlorine bleach-treated sewage (described below), and 187 strains were collected from surface water and groundwater samples.

**TABLE 1:** Presence of the *uspC*-IS*30-flhDC* marker in E. coli isolates or populations from various animal and environmental sources

Animal, human, or environmental source of E. coli isolates	No. of isolates tested	No. (%) of isolates with IS*30* element in *uspC-flhDC* intergenic region
Bovine	120	0 (0)
Bovine (*in silico*)^*a*^	41	0 (0)
Cat	21	0 (0)
Dog	61	0 (0)
Deer	48	0 (0)
Goose	54	0 (0)
Human	105	0 (0)
Human (*in silico*)^*a*^	1,107	0 (0)
Chicken	59	0 (0)
Moose	14	0 (0)
Muskrat	56	0 (0)
Horse	44	0 (0)
Pig	49	0 (0)
Coyote	44	0 (0)
Gull	18	0 (0)
Beaver	40	0 (0)
Sheep	47	0 (0)
Other animals (*in silico*)^*a*^^,^^*b*^	29	0 (0)
Wastewater (chlorine treated)^*c*^	70	41 (59)
Wastewater (raw)^*d*^	319	16 (5)
Surface water^*e*^	187	1 (0.5)

a “*In silico*” refers to BLAST sequence analysis of E. coli genomes in GenBank.

b Includes chickens and pigs.

c Chlorine-treated E. coli isolates originated from four different wastewater treatment plants in Alberta, Canada.

d Raw wastewater isolates were collected from various wastewater plants in Alberta (post-grit removal).

e Isolates from surface water were collected from various rivers and lakes in Alberta and were represented by one isolate per source.

Animal E. coli isolates were sourced from three bacterial libraries originally established from the following geographically disparate areas of Canada: (i) the Hamilton/Toronto region of Ontario, as described by Edge and Hill (23); (ii) Ottawa (Ontario), Lennoxville (Quebec), and Brandon (Manitoba), as described by Lyautey et al. (24); and (iii) Alberta, as described by White et al. (25) (bovine isolates) and Zhi et al. (20) (human isolates). For further details regarding bacterial libraries, please refer to the work of Zhi et al. (20).

For E. coli isolates from surface water and groundwater samples, the water samples were processed by membrane filtration and incubated on X-Gluc (5-bromo-4-chloro-3-indolyl-β-d-glucuronic acid; Dalynn, Calgary, Canada) agar plates at 44.5°C for 18 to 24 h. One blue colony was picked for each sample, streaked on MacConkey (Dalynn, Calgary, Canada) agar, and then incubated at 35°C for 18 to 24 h for further isolation. All presumptive human, animal, surface water, and groundwater E. coli isolates were confirmed as E. coli through biochemical analysis using a Vitek bacterial identification system (bioMérieux Canada Inc., St. Laurent, Canada) according to the manufacturer's instructions and protocols at the Provincial Laboratory for Public Health (ProvLab) in Edmonton, Alberta, Canada.

E. coli isolates from wastewater were obtained from a separate study involving an evaluation of ColiTag as a suitable growth medium for E. coli, with methods performed according to the U.S. Environmental Protection Agency (U.S. EPA) alternate test procedure (ATP) (26). This procedure incorporates a step in which bacteria are treated with chlorine bleach in order to stress the bacterial population. Briefly, raw sewage samples from different sewage treatment plants in Alberta, Canada, were collected and sent to ProvLab for analysis. Each raw sewage sample was treated with 3% sodium hypochlorite until the free chlorine residual concentration reached 0.3 to 0.5 ppm, causing an ∼2- to 4-log_10_ reduction in the culturable concentration of E. coli, according to the ATP protocol (26). Chlorine reactivity was neutralized by addition of a 10% solution of sodium thiosulfate. Chlorine-treated wastewater samples were then used to inoculate either ColiTag or lauryl Trypticase broth (LTB)/BCG medium according to method 9221.F in *Standard Methods for the Examination of Water and Wastewater* (27). E. coli was isolated from positive cultures in ColiTag or LTB/BCG medium by selective plating onto X-Gluc agar plates (28) and was incubated at 44.5°C for 24 h. Blue colonies were picked, streaked onto nonselective blood agar plates, and incubated at 35°C for 24 h. All presumptive wastewater E. coli isolates were confirmed through biochemical analysis at ProvLab by use of a Vitek bacterial identification system (bioMérieux Canada Inc., St. Laurent, Canada) according to the manufacturer's instructions and protocols. From this large collection of chlorine-tolerant E. coli isolates, we chose 70 isolates originating from four geographically separated wastewater treatment plants (WWTPs) for further phenotypic and genetic analyses.

### Environmental water samples.

In addition to the four WWTPs from which chlorine-tolerant E. coli strains were isolated and the microbes genetically characterized, wastewater samples (treated and untreated) were also collected from the City of Calgary, at two WWTPs (Bonnybrook and Pine Creek facilities). City of Calgary wastewater samples were used to (i) determine what proportion of wastewater samples (i.e., the entire population of E. coli in that water sample) possessed a novel genetic marker, *uspC*-IS*30-flhDC* (identified in this study and described below), at different points in the treatment process; and (ii) what proportion of the total number of E. coli organisms present in untreated wastewater possessed the *uspC*-IS*30-flhDC* marker. In the first analysis, 100 ml of wastewater (untreated and treated) was cultured in Colilert vessels (Idexx Laboratories, Inc., Westbrooke, ME) and incubated at 35°C for 24 h to enrich the population of E. coli in the sample. Samples positive for E. coli by Colilert testing were saved, and 1 ml of Colilert culture was centrifuged at 6,300 × *g* for 10 min to pellet bacteria for DNA extraction and PCR detection of the *uspC*-IS*30-flhDC* marker (see below). In experiments aimed at determining what proportion of E. coli isolates in a sample carried the marker, eight untreated wastewater samples were collected from the City of Calgary at different dates and at the two WWTPs, and the total E. coli count was quantified using 10-fold serial dilutions of the samples and most-probable-number (MPN) counting in a Colilert QuantiTray format (Idexx Laboratories, Inc.). The total number of E. coli organisms in a sample was determined based on the number of wells having both β-glucosidase (yellow) and β-glucuronidase (fluorescence) activities. To determine the number of E. coli organisms possessing the *uspC*-IS*30-flhDC* marker in that sample, the contents of each E. coli-positive well in the QuantiTray were aseptically removed with a syringe and transferred to a 2-ml centrifuge tube. Samples were centrifuged at 10,000 × *g* for 10 min to collect bacterial pellets. DNA was extracted according to the methods described below, and PCR was performed as described below. The number of E. coli-positive wells (yellow and fluorescent) and the number of those wells that were PCR positive for the *uspC*-IS*30-flhDC* marker for all eight wastewater samples were used to estimate the number of E. coli organisms in wastewater that were marker positive.

Environmental water samples also included groundwater and surface water samples. Groundwater samples were collected through routine testing at ProvLab. Drinking water samples from privately owned groundwater wells were tested for E. coli at ProvLab by use of Colilert testing (Idexx Laboratories Inc., Westbrooke, ME). Groundwater samples positive for E. coli by Colilert testing were saved, and 1 ml of Colilert culture was centrifuged at 10,000 × *g* for 10 min to collect bacterial pellets for DNA extraction (described below) and PCR detection of the *uspC*-IS*30-flhDC* marker (described below). Colilert test-positive samples represented the E. coli population present in the groundwater. Surface water samples were also obtained through routine submissions to ProvLab and processed by membrane filtration on X-Gluc agar plates (28) or by Colilert testing. From X-Gluc agar plates positive for E. coli, one presumptive E. coli colony was picked and verified as E. coli by use of a Vitek bacterial identification system (bioMérieux Canada Inc., St. Laurent, Canada), and colony PCR was performed to determine the occurrence of the *uspC*-IS*30-flhDC* marker (described below). Other surface water samples were processed by Colilert presence/absence testing (similarly to groundwater samples, as described above) in order to assess the prevalence of the *uspC*-IS*30-flhDC* marker in the E. coli populations in surface water samples.

### Phenotypic stress response (RpoS) activity.

The *rpoS*-regulated stress response was evaluated in individual E. coli isolates by using glycogen and catalase tests as described by White et al. (25). E. coli was grown on LB agar, and a single colony was transferred to tryptic soy broth (TSB) and grown overnight at 35°C. One microliter of the culture was inoculated onto LB agar for colony growth, and the plate was incubated at 28°C for 48 h. For detection of glycogen production, 5 ml of iodine solution (0.1 M I_2_, 0.03 M KI) was added to each plate, and the plates were allowed to stand for 5 min (25, 29). Dark brown colonies were considered positive, while pale brown or white colonies were considered attenuated or null for glycogen production (30, 31). Catalase activity was tested by applying a 6% (wt/vol) hydrogen peroxide solution to individual colonies, and a vigorous bubbling reaction was considered positive for catalase production. RpoS-positive (Salmonella enterica serovar Typhimurium ATCC 14028) (25) and RpoS-negative (S. enterica serovar Typhimurium Δ*rpoS*) (25) isolates were used as control strains. E. coli isolates were considered RpoS positive if both the glycogen and catalase tests were positive.

### DNA extraction from individual E. coli isolates and cultured water samples.

All E. coli isolates used in this study were grown in TSB, and genomic DNAs were extracted from E. coli cultures by use of DNeasy Blood & Tissue kits (Qiagen, Toronto, Ontario, Canada) according to the manufacturer's instructions. Genomic DNAs were then stored at −20°C. For wastewater and raw water samples testing positive for E. coli by Colilert testing or membrane filtration (as described above), the DNAs from bacteria were extracted by suspending the pellets in boiling molecular biology-grade water for 10 min, followed by centrifugation at 10,000 × *g* for 10 min. The supernatant containing each DNA was then stored at −20°C until it was used for PCR.

### Detection of virulence genes in wastewater E. coli isolates.

Ten E. coli isolates possessing the wastewater-specific *uspC*-IS*30-flhDC* marker were tested for 28 virulence genes associated with E. coli pathogenesis in humans and animals. Complete lists of virulence genes, primers, and conditions are provided in Tables 2 and 3. The 10 isolates selected were from four different wastewater treatment plants in Alberta, Canada. Real-time PCR was used to detect Shiga toxin 1 (*stx*_1_) and Shiga toxin 2 (*stx*_2_), while all other virulence genes were amplified using endpoint PCR. For all real-time PCRs, the reaction mixture contained 10 μl TaqMan Fast Advanced master mix (Applied Biosystems, Foster City, CA), 0.9 μM (each) primers, 0.25 μM TaqMan probe, 5 μl DNA template, and molecular biology-grade water added to a total volume of 20 μl. The real-time PCR conditions were 50°C for 2 min and 95°C for 30 s followed by 40 cycles of 95°C for 3 s and 60°C for 30 s. For all endpoint PCRs, the reaction volume contained 5 μl of E. coli genomic DNA template, 12.5 μl of Fermentas Maxima Hotstart 2× master mix (ThermoFisher Scientific, Waltham, MA, USA), 0.5 μM (each) primers, and molecular biology-grade water added to a total volume of 25 μl. The PCR products were run in a 2% agarose gel in 1× Tris-EDTA-acetate (TAE) buffer (Promega, Madison, WI) at 140 V for 45 min.

**TABLE 2:** PCR primers for amplification of virulence genes

Primer set	Gene target	Primer name	Primer sequence (5′-3′)	Product size (bp)	Reference
I	*aer*	aer-F	TACCGGATTGTCATATGCAGACCGT	601	25
		aer-R	AATATCTTCCTCCAGTCCGGAGAAG		
	*papC*	papC-F	GTGGCAGTATGAGTAATGACCGTTA	202	
		papC-R	ATATCCTTTCTGCAGGGATGCAATA		
	*traT*	traT-F	GGTGTGGTGCGATGAGCACAG	287	
		traT-R	CACGGTTCAGCCATCCCTGAG		
II	PAI	PAI-F	GGACATCCTGTTACAGCGCGCA	921	25
		PAI-R	TCGCCACCAATCACAGCCGAAC		
	*fimH*	fimH-F	TGCAGAACGGATAAGCCGTGG	505	
		fimH-R	GCAGTCACCTGCCCTCCGGTA		
III	*iroN*	iroN-F	AAGTCAAAGCAGGGGTTGCCCG	667	25
		iroN-R	GACGCCGACATTAAGACGCAG		
	*iutA*	iutA-F	GGCTGGACATCATGGGAACTGG	301	
		iutA-R	CGTCGGGAACGGGTAGAATCG		
	*ibeA*	ibeA-F	AGGCAGGTGTGCGCCGCGTAC	169	
		ibeA-R	TGGTGCTCCGGCAAACCATGC		
IV	*cnf1*	cnfl-F	AAGATGGAGTTTCCTATGCAGGAG	497	25
		cnfl-R	CATTCAGAGTCCTGCCCTCATTATT		
	*papGII*	papGII-F	GGGATGAGCGGGCCTTTGAT	189	
		papGII-R	CGGGCCCCCAAGTAACTCG		
V	*fuyA*	fuyA-F	TGATTAACCCCGCGACGGGAA	784	25
		fuyA-R	CGCAGTAGGCACGATGTTGTA		
	*papGIII*	papGIII-F	GGCCTGCAATGGATTTACCTGG	257	
		papGIII-R	CCACCAAATGACCATGCCAGAC		
VI	*sfa foc*	sfa/foc-F	CTCCGGAGAACTGGGTGCATCTTAC	407	25
		sfa/foc-R	CGGAGGAGTAATTACAAACCTGGCA		
	*hlyA*	hlyA-F	AACAAGGATAAGCACTGTTCTGGCT	1,176	
		hlyA-R	ACCATATAAGCGGTCATTCCCGTCA		
VII	*iha*	iha-F	CTGGCGGAGGCTCTGAGATCA	826	25
		iha-R	TCCTTAAGCTCCCGCGGCTGA		
VIII	*aidA*	AIDA-I-F	TGCAAACATTAAGGGCTCG	370	78
		AIDA-I-R	CCGGAAACATTGACCATACC		
	*aidA*	aidA-F	CAGTTTATCAATCAGCTCGGG	450	
		aidA-R	CCACCGTTCCGTTATCCTC		
	*aah*	aah-F	CTGGGTGACATTATTGCTTGG	543	
		aah-R	TTTGCTTGTGCGGTAGACTG		
IX	LT	LTA-F	GGCGACAGATTATACCGTGC	696	78
		LTA-R	CCGAATTCTGTTATATATGTC		
	*stb*	STb-F	ATCGCATTTCTTCTTGCATC	172	
		STb-R	GGGCGCCAAAGCATGCTCC		
X	*hra*	hra-F	CAGAAAACAACCGGTATCAG	257	78
		hra-R	ACCAAGCATGATGTCATGAC		
XI	*eaeA*	eaeA-F	GACCCGGCACAAGCATAAGC	384	78
		eaeA-R	CCACCTGCAGCAACAAGAGG		
XII	*sta*	Sta-F	TCTTTCCCCTCTTTTAGTCAG	166	78
		Sta-R	ACAGGCAGGATTACAACAAAG		
XIII	*ipaH*	ipaH-III	GTTCCTTGACCGCCTTTCCGATACCGTC	600	78
		ipaH-IV	GCCGGTCAGCCACCCTCTGAGATAC		
XIV	*usp*	usp-F	CGGCTCTTACATCGGTGCGTTG	614	25
		usp-R	GACATATCCAGCCAGCGAGTTC		
XV	*irp2*	irp2-F	AAGGATTCGCTGTTACCGGAC6	285	25
		irp2-R	TCGTCGGGCAGCGTTTCTTCT		
XVI^*a*^	*stx*_1_	stx1-F	CATCGCGAGTTGCCAGAAT	78	79
		stx1-R	GCGTAATCCCACGGACTCTTC		
		stx1-P	FAM-CTGCCGGACACATAGAAGGAAACTCATCA-TAMRA		
XVII^*a*^	*stx*_2_	stx2-F	CCGGAATGCAAATCAGTC	113	79
		stx2-R	CAGTGACAAAACGCAGAACT		
		stx2-P	FAM-ACTGAACTCCATTAACGCCAGATATGA-TAMRA		

a Probe sequences (stx1-P and stx2-P) are also listed. FAM, 6-carboxyfluorescein; TAMRA, 6-carboxytetramethylrhodamine.

**TABLE 3:** PCR conditions for amplification of virulence genes

Primer set(s)^*a*^	Initial denaturation		No. of cycles	Cycling conditions						Final extension	
				Denaturation		Annealing		Extension			
	Temp (°C)	Time (min)		Temp (°C)	Time (s)	Temp (°C)	Time (s)	Temp (°C)	Time (s)	Temp (°C)	Time (min)
I to VII, XIV, XV	94	5	33	94	30	63	30	72	60	72	7
VIII	94	5	33	94	60	63	60	72	30	72	10
IX	94	5	30	94	60	58	60	72	60	72	7
X	94	5	33	94	30	55	30	72	30	72	7
XI	94	5	30	94	60	58	60	72	40	72	7
XII	94	5	30	94	60	53	60	72	30	72	7
XIII	94	5	33	94	30	50	30	68	60	72	7

a The primer sets in this table correspond to the primer sets listed in Table 2.

### Detection of an environmental resistance genomic island in wastewater E. coli isolates.

The presence of a heat resistance genomic island, known as the locus of heat resistance (LHR) and previously identified in highly heat-resistant E. coli strains (32), was also assessed in chlorine-tolerant wastewater strains. PCRs targeting three fragments of this genomic island were performed on all 70 wastewater E. coli isolates. PCR primers for the LHR are listed in Table 4. PCR conditions for fragment A were as follows: initial denaturation at 95°C for 5 min, 30 cycles of 95°C for 45 s, annealing at 63°C for 1 min, and 72°C for 1.5 min, and a 7-min extension at 72°C. PCR conditions for fragment B were as follows: initial denaturation at 95°C for 5 min, 30 cycles of 95°C for 45 s, annealing at 64°C for 1 min, and 72°C for 2.5 min, and a 7-min extension at 72°C. PCR conditions for fragment C were the same as the conditions for fragment B except that the annealing temperature was 58°C. The PCR products for fragments A, B, and C were run in a 2% agarose gel in 1× TAE buffer (Promega, Madison, WI) at 140 V for 45 min.

**TABLE 4:** PCR primers targeting the intergenic regions and the LHR

Target	Primer	Primer sequence (5′-3′)	Reference
*asnS-ompF*	ompF-F	TACGTGATGTGATTCCGTTC	77
	ompF-R	TGTTATAGATTTCTGCAGCG	
*csgBAC-csgDEFG*	csgD-1	GGACTTCATTAAACATGATG	77
	csgD-2	TGTTTTTCATGCTGTCAC	
*uspC-flhDC*	flhDC-F	GAGGTATGCATTATTCCCACCC	25
	flhDC-R	TGGAGAAACGACGCAATC	
*uspC*-IS*30-flhDC*	flh-IS-F	CGGGGAACAAATGAGAACAC	This study
	flh-IS-R	TGGAGAAACGACGCAATC	
LHR fragment A	HR-F1	TTAGGTACCGCTGTCCATTGCCTGA	32
	HS-R1	AGACCAATCAGGAAATGCTCTGGACC	
LHR fragment B	HR-F2.2	GAGGTACCTGTCTTGCCTGACAACGTTG	32
	HR-R2	TATCTAGAATGTCATTTCTATGGAGGCATGAATCG	
LHR fragment C	HS-F1	GCAATCCTTTGCCGCAGCTATT	32
	HR-R3	GTCAAGCTTCTAGGGCTCGTAGTTCG	

### Genetic characterization of E. coli isolates obtained from humans, animals, and wastewater. (i) Phylogrouping.

Phylogrouping of E. coli isolates was performed by using PCR assays developed by Clermont et al. (33). The PCR assays targeting *chuA*, *yjaA*, and the TSPE4.C2 loci were adopted from a previous version of this phylogrouping method (34). The total volume for all PCR mixtures was 25 μl, with each mixture containing 5 μl of E. coli genomic DNA template, 12.5 μl of Fermentas Maxima Hotstart 2× master mix (ThermoFisher Scientific), 1.25 μl of each primer (10 pmol/μl), and 5 μl molecular biology-grade water. Each PCR mixture contained 1.25 U of *Taq* polymerase, a 200 μM concentration of each deoxynucleoside triphosphate (dNTP), 1× PCR buffer, and 2 mM Mg^2+^. The PCR conditions used for phylogrouping were the same as those described previously (33, 34).

### (ii) Phylogenetic analysis of human, animal, and wastewater isolates.

For evaluation of genetic similarities among human, animal, and wastewater E. coli isolates, we used a maximum likelihood phylogenetic analysis of the following three intergenic regions: (i) *uspC-flhDC*, (ii) *csgBAC-csgDEFG*, and (iii) *asnS-ompF*. The selection of these regions was based on our previous publications, in which host-specific SNP biomarkers were identified in these three intergenic regions across 15 animal species (20) and these intergenic sequences correlated with adaptive phenotypes in E. coli (25). The PCR conditions for assays targeting the *uspC-flhDC*, *csgBAC-csgDEFG*, and *asnS-ompF* intergenic regions were as follows: initial denaturation at 95°C for 4 min, 33 cycles of 95°C for 30 s, 58°C for 30 s, and 72°C for 1 min, and a 7-min extension at 72°C. Electrophoresis was performed on a 2% agarose gel in 1× TAE buffer at 140 V for 45 min. Primers for the PCR assays are listed in Table 4.

The PCR products were purified using an Agencourt AMPure XP PCR purification kit (Beckman, Brea, CA). DNA sequencing was performed bidirectionally by Macrogen Inc. (Seoul, South Korea). The ClustalX 2.0 (35) program was used for sequence alignment, and the resulting alignments were manually edited to trim the 5′ and 3′ regions with missing data.

Due to the presence of unique DNA insertion elements in chlorine-tolerant wastewater E. coli isolates within the *uspC-flhDC* intergenic region (described below), we based our phylogenetic analysis on the unrelated *csgBAC-csgDEFG* and *asnS-ompF* intergenic regions. The sequences were assembled into a concatenated single file for each E. coli strain, aligned, and analyzed by RAxML (36) to generate a maximum likelihood phylogenetic tree based on the gamma-plus-Invar model (36). The tree was edited to map to different groupings by using the Interactive Tree of Life (iTOL) online tool (37, 38).

### (iii) SNP biomarker analysis.

As an alternative method for evaluating the genetic uniqueness of chlorine-tolerant wastewater E. coli isolates, we used a novel logic regression-based biomarker approach described by Zhi et al. (20) to identify SNP patterns in intergenic regions (*csgBAC-csgDEFG* and *asnS-ompF*) that were highly specific to wastewater E. coli isolates. This entailed using logic regression as a supervised learning classification method for distinguishing E. coli isolates from different animal host/nonhost environmental reservoirs by using host-specific informative SNP patterns in intergenic DNA sequences. We sought to determine whether a highly specific SNP pattern existed for chlorine-tolerant wastewater E. coli isolates and, if so, if it was distinct from those for other human and animal isolates. In this analysis, biomarker sensitivity was defined as the proportion of samples from a targeted host or wastewater sample that carried a specific SNP pattern. Biomarker specificity was defined as the proportion of samples from host/nonhost environments other than the target host/nonhost environment (i.e., all other human and animal hosts) that did not carry the SNP biomarker of interest. Model building and statistical evaluations were done according to the method of Zhi et al. (20).

Since logic regression attempts to fit a very flexible model to a set of observations, overfitting, a common problem in supervised learning, is a concern. We used a 5-fold cross-validation as an attempt to prevent overfitting and to unbiasedly evaluate the performance of the final logic SNP biomarker models for human, animal, and wastewater E. coli isolates, as outlined previously (20). For 5-fold cross-validation, data sequences from each host/nonhost environment were randomly divided (i.e., by computer-based randomization) into five subsets with equal numbers of samples. Four subsets of data were used as training data for logic regression analysis, and the last data subset was used as testing data to evaluate the logic regression model that was identified based on the training data. This was repeated for all possible subsets for training and testing, and the results were averaged over the five subsets.

### Development of a PCR assay specific to naturalized wastewater E. coli isolates.

During the genetic screening and comparison of human, animal, and wastewater E. coli isolates, we observed that many of the wastewater isolates surviving chlorine bleach treatment contained an insertional sequence (IS*30*) element located specifically in the *uspC-flhDC* intergenic region (herein referred to as the *uspC*-IS*30-flhDC* marker). As such, we sought to develop a sensitive and specific endpoint PCR to target the IS*30* element in the *uspC-flhDC* intergenic region. Primers were designed (Table 4) and tested against genomic extracts of chlorine-tolerant wastewater E. coli isolates. The PCR conditions used to amplify the *uspC*-IS*30-flhDC* marker were as follows: initial denaturation at 95°C for 4 min, 35 cycles of 95°C for 30 s, 60°C for 30 s, and 72°C for 30 s, and a 7-min extension at 72°C.

To test the PCR sensitivity, a plasmid containing the genetic marker was constructed. Specifically, genomic DNA from a chlorine-tolerant E. coli isolate containing the *uspC*-IS*30-flhDC* marker was used as a DNA template, and PCR was used to amplify the *uspC-flhDC* intergenic region (primers flh-IS-F and flh-IS-R) (Table 4). The PCR product was resolved in a 2% agarose gel in 1× TAE buffer (Promega) at 140 V for 45 min and then purified from the gel by use of a QIAquick gel extraction kit (Qiagen), and the amplicon was cloned using a TOPO TA cloning kit (Invitrogen). Isolation of recombinant plasmid DNA was performed using a QIAprep miniprep kit (Qiagen), and the presence of the correct insert was confirmed by PCR screening and DNA sequencing of the cloned inserts. DNA sequencing was performed by Macrogen Inc. (Seoul, South Korea).

PCR sensitivity was tested using both cloned plasmid DNA and E. coli genomic DNA. The concentrations of plasmid and genomic DNAs were quantified using a Qubit 2.0 fluorometer (Invitrogen). The plasmid copy number was determined from cloned targets, and the number of targets in the genome was calculated by assuming a genome size of 4.7 Mbp and a single copy of each gene within each genome. Tenfold serial dilutions of both plasmid and genomic DNAs were made in replicates of eight, and the *uspC*-IS*30-flhDC* marker was amplified by PCR. The limit of detection (with 95% confidence intervals [LOD_95_]) was calculated using Excel (39).

### Evaluating the specificity of *uspC*-IS*30-flhDC* PCR.

The specificity of the PCR primers targeting the *uspC*-IS*30-flhDC* marker in chlorine-tolerant wastewater E. coli isolates was evaluated against DNAs obtained from (i) 845 E. coli isolates from humans and animals, to determine if the IS*30* element in the *uspC-flhDC* intergenic region was specific to chlorine-tolerant wastewater E. coli; and (ii) 178 environmental water samples (E. coli-positive surface water, groundwater, and wastewater samples), to determine the prevalence of the marker in E. coli populations from wastewater compared to that in E. coli populations from groundwater and surface water sources.

## RESULTS

### Evidence of genetically unique strains of chlorine-tolerant E. coli in wastewater.

The *uspC-flhDC* intergenic region was amplified from 824 of 845 E. coli isolates from 15 different animal species, with all of them generating a PCR amplicon that was 739 bp long. In comparison, when the same PCR was performed on 70 E. coli strains isolated from chlorine-treated wastewater samples, 43 of the 70 isolates (61.4%) produced PCR amplicons of 1,223 or 1,155 bp. The abnormally long PCR products were sequenced bidirectionally by Sanger sequencing and aligned with the sequences of E. coli isolates from human and animals, and two large insertion sequences were found in the *uspC-flhDC* intergenic region of these isolates. These large internal sequences were analyzed by BLAST searches and searches against the IS Finder Database (41). Fifty-nine percent (41/70 isolates) of the chlorine-tolerant wastewater isolates contained an insertional sequence element designated the IS*30* element (40), while 3% (2/70 isolates) contained another insertional element sequence, ISEc33 (42). PCR amplicons of 1,223 bp were shown to contain the IS*30* element, whereas wastewater E. coli isolates possessing the ISEc33 element produced amplicons of 1,155 bp. The *IS30* element is one of the smallest known bacterial transposons encoding a motility gene transposase (40).

The entire *uspC*-IS*30-flhDC* sequence was processed through BLAST analysis in GenBank, and no similar sequences were identified in the NCBI database, suggesting that the positional location of the IS*30* element within the *uspC-flhDC* intergenic region may be unique to chlorine-tolerant wastewater E. coli isolates. As further evidence of this, none of the human or animal E. coli isolates analyzed in this study possessed the position-specific IS*30* element (Table 1). Furthermore, BLAST analysis of the entire *uspC*-IS*30-flhDC* sequence against E. coli genomes from isolates originating from humans (*n* = 1,107), cattle (*n* = 41), and other animals (*n* = 29) (Table 1) revealed no sequence similarities in these genomic databases.

**FIG 1:**
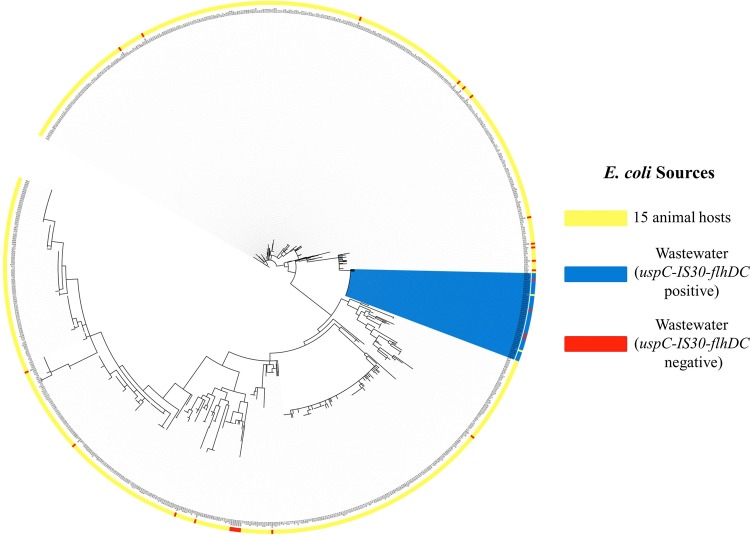
Unrooted maximum likelihood phylogenetic tree encompassing 848 E. coli strains obtained from chlorine-treated wastewater (68 isolates) and 15 animal host groups (780 isolates) (Table 2) and based on an analysis of the concatenated DNA sequences of two intergenic regions (*csgBAC-csgDEFG* and *asnS-ompF*). Of the 70 chlorine-tolerant wastewater E. coli isolates, two failed to produce PCR products for the *csgBAC-csgDEFG* intergenic region. The colored ring that overlays the unrooted maximum likelihood tree indicates different E. coli groups, as defined by (i) human/animal host sources (yellow), (ii) wastewater isolates possessing the *uspC*-IS*30-flhDC* marker (blue), and (iii) wastewater isolates not possessing the *uspC*-IS*30-flhDC* marker (red). Note that all chlorine-tolerant wastewater isolates that possessed the *uspC*-IS*30-flhDC* marker (and which were isolated from 4 different WWTPs) grouped together within a single clade.

In order to provide further evidence of the genetic uniqueness of these wastewater isolates, we performed a maximum likelihood phylogenetic analysis as well as logic regression biomarker analysis (20) of intergenic regions (*csgBAC-csgDEFG* and *asnS-ompF*) unrelated to the *uspC-flhDC* region for all human, animal, and wastewater isolates. Of the 70 chlorine-tolerant wastewater E. coli isolates, 68 were amplifiable at both the *csgBAC-csgDEFG* and *asnS-ompF* regions. We sequenced the *csgBAC-csgDEFG* and *asnS-ompF* intergenic regions of these E. coli isolates as well as 780 E. coli isolates from 15 different animal species (Table 1). Intergenic sequence data were concatenated, and a multiple-sequence alignment was then used to construct a maximum likelihood phylogenetic tree (Fig. 1). All chlorine-tolerant wastewater E. coli isolates possessing the IS*30* element in the *uspC-flhDC* intergenic region clustered together in the *csgDEFG*/*asnS-ompF*-derived phylogenetic tree, providing evidence for their genetic difference from animal and human strains.

Microbial host-specific biomarker analysis (20) was also used to determine genetic uniqueness based on SNP patterns in DNA sequences of E. coli that relate to animal host origin or nonhost environmental origin. Logic regression analysis of SNP patterns in the concatenated *csgDEFG*/*asnS-ompF* intergenic sequences revealed host-specific patterns across most human and animal E. coli isolates (Table 5). For human E. coli isolates, for which logic regression models were subjected to 5-fold cross-validation, 41% of isolates carried a SNP biomarker pattern that was unique to humans (i.e., specificity of 1) (Table 5). Other animals for which a sensitive host-specific biomarker could be found (i.e., >35% of isolates in the animal group carried the specific biomarker, and the model was subjected to 5-fold cross-validation) included deer (37%), chickens (56%), muskrats (77%), coyote (59%), beaver (35%), and sheep (37%), all with specificities of ≥99%. Remarkably, one of the greatest sensitivities observed in SNP biomarker analysis was for the chlorine-tolerant wastewater E. coli group, with 82% of the 68 isolates possessing a SNP biomarker with very high specificity to wastewater (≥99%) based on grouped analysis (Table 5). Of the 41 chlorine-tolerant E. coli isolates possessing the IS*30* element in the *uspC-flhDC* intergenic region, all possessed the same SNP biomarker unique to wastewater in the *csgDEFG*/*asnS-ompF* intergenic sequences, providing further evidence of a common genetic background among the wastewater strains that is genetically different from that of human and animal isolates.

**TABLE 5:** Logic regression-based SNP analysis of E. coli samples classified according to isolation source

Source	No. of samples	Logic regression		Fivefold cross-validation	
		Sensitivity	Specificity	Sensitivity	Specificity
Wastewater^*a*^	68	0.82	1	0.76	0.99
Bovine	120	0.38	0.98	0.21	0.96
Cat	21	0.29	1	0	1
Dog	61	0.57	0.96	0.23	0.97
Deer	48	0.88	0.95	0.37	1
Goose	54	0.24	0.99	0.09	0.99
Human	105	0.46	1	0.41	1
Chicken	59	0.68	1	0.56	1
Moose	14	0.29	0.99	0	1
Muskrat	56	0.77	0.99	0.77	0.99
Horse	44	0.11	0.99	0	1
Pig	49	0.39	1	0.22	1
Coyote	44	0.64	0.99	0.59	0.99
Gull	18	0.44	0.99	0.05	0.99
Beaver	40	0.45	0.99	0.35	1
Sheep	47	0.49	1	0.37	1

a Analysis was performed on the *csgBAC-csgDEFG* (listed as “csg” in the model) and *asnS-ompF* (listed as “omp” in the model) intergenic regions as described in Materials and Methods. The logic regression-based biomarker model for E. coli isolates from wastewater was as follows: logic *E*[*Y*] = 3.33 − 19.9 × {(omp47_T or csg1122_G) or [(not omp564_C) or (not omp335_A)]} − 7.01 × {[(not csg1180_G) and omp440_T and (not omp81_T)] and [csg993_G and omp481_G and (not omp423_A)]}. In this wastewater logic regression model, *E*[*Y*] is the probability of an E. coli isolate originating from a wastewater source. In reading the model output, a value designated “omp47_T” refers to the thymine base at nucleotide position 47 in the multisequence alignment of the *asnS-ompF* intergenic region. For further interpretation of model variables, see the work of Zhi et al. (20).

A PCR-based phylogrouping method was also used to characterize all 70 chlorine-tolerant E. coli wastewater isolates, including those that did not contain either the IS*30* or ISEc33 element. Phylogroup A was the most prevalent group (74.3% [52/70 isolates]), followed by groups D (11.4% [8/70 isolates]), B1 (7.1% [5/70 isolates]), B2 (4.3% [3/70 isolates]), and E (2.9% [2/70 isolates]). Notably, all chlorine-tolerant wastewater E. coli strains that carried the IS*30* element in the *uspC-flhDC* region belonged to phylogroup A.

To evaluate whether the chlorine-tolerant wastewater E. coli strains were simply adapted to survival in water, we examined 187 E. coli isolates collected from various other water sources (rivers, lakes, etc.) for the presence of the IS*30* element in the *uspC-flhDC* intergenic sequence. Only 1 of the 187 E. coli isolates (0.5%) collected from surface water was positive, suggesting that strains possessing the *uspC*-IS*30-flhDC* sequence may be adapted specifically for wastewater survival. This was further supported by the observation that the prevalence of the IS*30* element in the *uspC-flhDC* region was far greater (59% [41/70 isolates]) in E. coli isolates obtained from chlorine-treated sewage than in E. coli isolates obtained directly from untreated sewage (5% [16/319 isolates]) (Table 1).

DNA sequence analysis, phylogenetic analysis, biomarker analysis, phylogrouping, and comparative prevalence assessments all supported the evidence that E. coli isolates that carry the IS*30* element in the *uspC-flhDC* region appear to be genetically distinct from E. coli strains isolated from humans and animals and may be adapted specifically to survival in wastewater. It is worth noting that the 41 wastewater isolates containing the IS*30* element in the *uspC-flhDC* region were actually isolated from four geographically segregated WWTPs in the province of Alberta. These data suggest not only that these strains are unique genetically but also that the genetic signature is consistent across geographic regions.

### Determination of RpoS stress response activity and presence of a heat resistance genomic island in wastewater E. coli isolates.

We sought to determine whether the chlorine-tolerant wastewater E. coli isolates possessing the *uspC*-IS*30-flhDC* marker also possessed the phenotypic properties of a generalized stress response (RpoS), which is important for environmental survival. The rationale was to determine whether the unique genetic background of these isolates correlated with known adaptive survival strategies for environmental persistence of E. coli. All 41 wastewater isolates possessing the *uspC*-IS*30-flhDC* marker were positive for both glycogen production and catalase activity, suggesting that these strains were phenotypically adapted to survive in the environment.

PCR amplifications of three genetic fragments from a heat resistance genomic island were performed on all 70 wastewater E. coli isolates. All 41 *uspC*-IS*30-flhDC*-positive E. coli isolates were positive for the three PCR fragments (A, B, and C) typically found in this large genomic island.

### Prevalence of virulence genes in wastewater E. coli isolates.

Ten *uspC*-IS*30-flhDC* marker-positive wastewater E. coli strains were analyzed for the presence of 28 virulence genes. Twenty-seven of the 28 virulence genes were not found in any of these wastewater E. coli strains, with the exception being the *fimH* gene; all 10 isolates were positive for *fimH*, encoding an adhesive protein localized at the tips of type 1 fimbriae.

### Evaluation of a PCR assay targeting the *uspC*-IS*30-flhDC* region in chlorine-tolerant wastewater E. coli isolates.

The high prevalence of the *uspC*-IS*30-flhDC* intergenic signature in chlorine-tolerant wastewater E. coli isolates and its apparent absence in the genomes of E. coli strains isolated from other human, animal, and even nonhost environmental sources (i.e., surface water) suggested that this sequence may be useful as a genetic marker of municipal wastewater contamination in the environment (i.e., untreated/inadequately treated sewage, discharge from combined sewer outfalls, sewage pipe breakage, or illicit sewage connections to storm water). Consequently, we developed a sensitive and specific endpoint PCR for detection of these wastewater E. coli strains. The PCR assay was designed to target the site-specific location of the IS*30* element in the *uspC-flhDC* intergenic region. The forward primer was identical to the primer used to amplify the *uspC-flhDC* intergenic region, but the reverse primer targeted the IS*30* sequence (Table 4). The position-specific PCR produced an amplicon of 386 bp. The LOD_95_ of the optimized PCR assay was 13 copies (95% confidence interval [CI_95_], 6 to 28 copies) based on cloned plasmid DNA templates, while the LOD_95_ for genomic DNA was 19 copies (CI_95_, 9 to 42 copies).

Because our previous work confirmed that the 845 animal and human E. coli isolates used in this study did not possess the IS*30* element inside the *uspC-flhDC* intergenic sequence (20), the specificity of the PCR was tested against only a random selection of 90 E. coli isolates. The rationale for testing specificity at this level was based on the fact that although the IS*30* element may exist in different areas of the genome in human and animal E. coli isolates, we wanted to determine whether its specific location in the *uspC-flhDC* intergenic region was unique to chlorine-tolerant wastewater E. coli isolates. The 386-bp PCR amplicon was not observed for any of the human and animal E. coli isolates tested (data not shown), whereas all of the 41 chlorine-tolerant wastewater E. coli isolates were positive by this endpoint PCR.

### Prevalence of the *uspC*-IS*30-flhDC* marker in environmental water samples.

**FIG 2:**
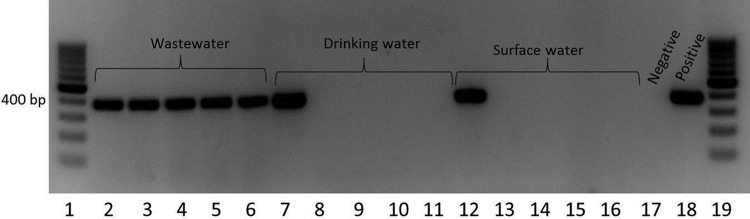
PCR amplification of the *uspC*-IS*30-flhDC* marker from Colilert-enriched E. coli-positive wastewater, drinking water, and surface water samples. Lanes 2 to 16 represent the PCR results for individual water samples. Lanes 1 and 19, molecular size marker (GeneRuler 100 bp; Thermo Scientific); lanes 2 to 6, wastewater; lanes 7 to 11, drinking water; lanes 12 to 16, surface water; lane 17, positive control (genomic DNA from an E. coli isolate possessing the *uspC*-IS*30-flhDC* marker); lane 18, negative control (genomic DNA from an E. coli isolate that did not possess the *uspC*-IS*30-flhDC* marker). The PCR amplicon obtained by targeting the *uspC*-IS*30-flhDC* marker is 386 bp long.

The *uspC*-IS*30-flhDC*-specific PCR assay was subsequently tested against environmental water samples in order to verify the presence of this marker in environmental wastewater samples: to evaluate the potential applicability of *uspC*-IS*30-flhDC* PCR for routine public health screening of water supplies, we evaluated the specificity and prevalence of this marker in wastewater (treated and untreated [City of Calgary WWTPs]), groundwater, and surface water (rivers, lakes, etc.) samples. Samples that were culture positive for E. coli (based on presence/absence testing by Colilert testing) were screened for the *uspC*-IS*30-flhDC* marker by PCR. The *uspC*-IS*30-flhDC* marker was found in 92% of 50 wastewater samples tested (untreated or treated) (Table 6 and Fig. 2), indicating a high prevalence of the strains in E. coli populations found in wastewater. All untreated wastewater samples from the City of Calgary's two WWTPs (*n* = 21 [100%]), as well as most secondarily treated samples (16/17 isolates [94%]) and UV-treated wastewater samples (9/12 isolates [75%]), were positive for E. coli strains carrying the *uspC*-IS*30-flhDC* marker. Among 57 E. coli culture-positive groundwater samples observed by Colilert testing, only 3 samples (5%) were positive for the *uspC*-IS*30-flhDC* marker, while of the 71 surface water samples that were positive for E. coli by Colilert culture, only 2 samples (3%) carried this marker. The high prevalence of this marker in E. coli populations in wastewater compared to E. coli populations in contaminated groundwater and surface water demonstrates that the *uspC*-IS*30-flhDC* marker appears to be specific to human municipal wastewater contamination.

**TABLE 6:** Prevalence of the *uspC*-IS*30-flhDC* marker in E. coli-positive surface water, drinking water, and wastewater samples

Source of E. coli-positive water samples	No. of samples	No. (%) of marker-positive samples
Wastewater (total)	50	43 (92)
Untreated wastewater (post-grit removal)	21	21 (100)
Secondarily treated wastewater	17	16 (94)
UV-treated wastewater	12	9 (75)
Surface water	71	2 (3)
Drinking water (groundwater)	57	3 (5)

## DISCUSSION

E. coli, although defined as a single species, shares only 10% of its pangenome among individual members (43). This genetic diversity, largely explained by an abundance of accessory genes, may account for the ability of E. coli to survive and adapt to various niches, including a diverse array of gastrointestinal microenvironments in warm-blooded animals as well as the nonhost environment. Naturalized strains of E. coli have been identified in various water sources (6–8), but to date, evidence for the presence of naturalized strains of E. coli in a wastewater environment has not been reported. We hypothesized that the intrinsic pressures imposed by wastewater treatment drive adaptation of certain strains of E. coli toward survival in wastewater and lead to the selection of naturalized endogenous populations of this bacterium in wastewater matrices and that these strains are genetically different from those found in human and animal hosts.

In order to evaluate this, we examined the population of E. coli strains present in untreated sewage that were chlorine tolerant. Sewage samples were collected from geographically segregated WWTPs in Alberta, Canada, and subjected to the U.S. EPA ATP protocols for evaluating recovery of chlorine-stressed organisms in selective media. Of the 70 E. coli isolates collected from chlorine-treated sewage in the laboratory (i.e., deemed chlorine tolerant), 59% possessed an IS*30* element within the intergenic region between the *uspC* and *flhDC* genes. The IS*30* element, a 1.2-kb DNA fragment typically found in various locations across the E. coli genome, was originally described by Caspers et al. (40) but, to date, has not been reported to occur within the intergenic region between the *uspC* and *flhDC* genes. The specific location of the IS*30* element in the *uspC-flhDC* intergenic region was unique to the chlorine-tolerant wastewater E. coli isolates, as we did not observe this positional location of the insertion sequence in a library of 845 E. coli isolates from 15 different animal species (including humans), in any sequences submitted to GenBank, or across 1,177 E. coli genomes for which whole-genome sequences were available from the NCBI (1,107 human isolates, 41 bovine isolates, and 29 isolates from other animals).

To further evaluate their genetic uniqueness, E. coli strains were subject to phylogrouping, phylogenetic analysis, and host-specific biomarker analysis (20). All chlorine-tolerant wastewater E. coli isolates possessing the *uspC*-IS*30-flhDC* marker belonged to phylogroup A. In a separate study (44), multilocus sequence typing (MLST) was used to identify a specific sequence type that was overrepresented by E. coli strains isolated from the nonhost environment. However, in contrast to our findings, these strains were assigned to phylogroup B1. Other studies have also identified the B1 phylogroup as being more prevalent for isolates collected from water (45, 46). However, it is interesting that isolates collected in these studies were obtained from surface water (i.e., rivers and lakes) as opposed to wastewater. In our study, only 1 of 187 E. coli isolates obtained from surface water sources possessed the *uspC*-IS*30-flhDC* marker. Even in this instance, it is possible that this isolate originated from wastewater, as it was collected downstream from a municipal wastewater treatment plant. Indeed, in agreement with our study, E. coli strains from phylogroup A were found to be more abundant in wastewater in other studies (47, 48).

In order to address whether the chlorine-tolerant strains may be genetically unique, maximum likelihood phylogenetic analysis and SNP biomarker analysis were carried out using DNA sequences at two alternate intergenic regions (*csgBAC-csgDEFG* and *asnS-ompF*) unrelated to the *uspC* and *flhDC* genes, and the data were compared across 780 human/animal E. coli isolates. Based on our previous studies examining these intergenic loci (20), traditional phylogenetic approaches failed to reveal host-specific clustering of animal and human E. coli strains, whereas biomarker analysis demonstrated a clear delineation in host-specific DNA biomarker patterns. Chlorine-tolerant wastewater E. coli isolates possessing the *uspC*-IS*30-flhDC* marker formed a single clade by traditional bioinformatics that was distinct from human and animal isolates, suggesting a common genetic background for these strains. SNP biomarker analysis at the same intergenic locus provided further evidence that these E. coli isolates were genetically unique. Logic regression-based biomarker analysis revealed that 82% of all chlorine-tolerant wastewater E. coli isolates possessed a SNP biomarker that was highly specific to wastewater and was not found in E. coli isolates collected from 15 different animal species, including humans. In addition, all of the 41 chlorine-tolerant wastewater isolates possessing the IS*30* element in the *uspC-flhDC* region also carried this wastewater-specific SNP biomarker in the *csgBAC-csgDEFG*/*asnS-ompF* intergenic regions.

It is particularly important that *uspC*-IS*30-flhDC*-carrying chlorine-tolerant E. coli strains were observed in a number of geographically separated WWTPs in Alberta, Canada. In fact, all wastewater treatment plants that we have tested to date possess E. coli strains containing the *uspC*-IS*30-flhDC* marker, suggesting a widespread dispersion of these strains and a functional role of the site-specific insertion of the IS*30* element in this intergenic region which seems to favor bacterial survival and persistence in a broad spectrum of municipal wastewater environments.

Phenotypically, all chlorine-tolerant strains possessing the *uspC*-IS*30-flhDC* marker are also positive for RpoS generalized stress response activity. The transcriptional regulator σ^S^ is a key regulator of the RpoS stress response in E. coli, initiating transcription of the genes essential for stress resistance (49–51). A resistance phenotype regulated by σ^S^ is the rdar phenotype (red, dry, and rough when grown on medium containing the dye Congo red), which enhances long-term survival under harsh conditions (52). Cells with the rdar phenotype secrete an extracellular matrix comprised of curli fimbriae, cellulose, and polysaccharides (53, 54), and the matrix provides increased resistance to disinfection (55, 56). Chiang et al. (57) observed that E. coli strains found in wastewater had the largest percentage of RpoS-positive E. coli strains compared to strains found in natural water, beach sand, and animal feces. In addition, a heat resistance genomic island was found in all *uspC*-IS*30-flhDC*-positive E. coli strains. This genomic island in E. coli, also called the locus of heat resistance (LHR), was reported in a recent study by Mercer et al. (32) and contains 16 open reading frames which are predicted to code for proteins that are associated with heat shock, cell envelope maintenance, and turnover of misfolded proteins (32). E. coli strains possessing the LHR have been shown to survive a heat shock temperature of 60°C for longer than 5 min (32). The LHR in E. coli is flanked by transposable elements, suggesting genetic mobility of the LHR among E. coli strains and raising food safety concerns about heat-resistant E. coli (32). Interestingly, the LHR in E. coli is homologous to a heat resistance plasmid observed in nosocomial pathogenic strains of Klebsiella pneumoniae (58). Bojer et al. (58, 59) demonstrated the conjugative transmissibility of the plasmid-mediated heat resistance phenotype to naive strains and the colocalization of heat resistance with antibiotic resistance determinants on these plasmids. Moreover, biofilm-producing K. pneumoniae cells have enhanced heat resistance compared to planktonic cells carrying the heat resistance plasmid, suggesting that biofilm formation and heat resistance promote environmental stability of these nosocomially persistent clones (60). It is extremely interesting that all chlorine-tolerant E. coli isolates carrying the *uspC*-IS*30-flhDC* locus also possessed the LHR. The fact that wastewater temperatures in Alberta fluctuate between 4°C and 20°C suggests that the LHR, and the proteins encoded therein, may have a broader spectrum of activity in promoting environmental resistance to more than just heat stress. The heat resistance characteristic in combination with RpoS stress response activity may contribute to the survival of these wastewater strains in harsh environments.

The IS*30* element, as well as other insertional elements, was previously found to alter gene expression and, consequently, bacterial phenotypes (61, 62). In one study, insertion of an IS element appeared to reduce transcriptional repression, resulting in increased expression of the *flhD* operon (63). It was also noted that several IS elements were located upstream of the *flhD* promoter in E. coli isolates that had a high swarming rate, while no IS elements were found in strains with poor motility (63). In turn, motility has been found to be associated with biofilm formation (64). Wood et al. (65) compared the biofilm formation capacities and motilities of several E. coli strains, and they demonstrated that strains with the greatest motility also had the highest biofilm formation capacity. Formation of a biofilm is a strategy often used by bacteria for survival in harsh environments (55, 56). Consequently, we hypothesize that the site-specific insertion of the IS*30* element in the *uspC-flhDC* region may alter flagellar expression, and consequently the motility and biofilm-forming capacity of these strains, ultimately leading to treatment resistance and environmental persistence of these strains in a wastewater environment and potentially complementing the RpoS generalized stress response and the environmental resistance phenotypes encoded by the LHR. At present, we do not have any function-based evidence that a causal relationship exists between carriage of the *uspC*-IS*30-flhDC* genetic marker and enhanced survival of E. coli under stressed conditions.

Interestingly, *uspC* and *flhDC* are divergently transcribed across the E. coli chromosome, and consequently the IS*30* element may also play a role in regulating transcription of *uspC* (also known as *yecG*). The universal stress protein superfamily, for which 6 proteins exist in E. coli (UspA, -C, -D, -E, -F, and -G), is involved in cellular stress responses to osmotic, oxidative, antibiotic, and UV-induced stressors (66, 67). More specifically, the *uspC* gene product has been shown to enhance flagellar production and motility in E. coli (67). Although other universal stress proteins have been shown to be important for dealing with oxidative stressors, such as hydrogen peroxide and superoxide (not specifically examined in this study), the *uspC* gene has also been shown to be important for enhancing UV resistance in E. coli (66). Both flagellar and universal stress response genes are known to be important virulence factors for pathogenic bacteria (68, 69). For example, mice infected with isogenic *uspA* mutants of S. enterica serovar Typhimurium have better survival outcomes (i.e., delayed death) than those infected with a pathogenic parent strain of the bacteria (70). Thus, in addition to possessing the RpoS and LHR stress-adaptive phenotypes/genotypes, the naturalized strains of E. coli in wastewater may also have an enhanced universal stress response.

It is important to mention that wastewater treatment processes at the City of Calgary's WWTPs incorporate oxidative aerobic digestion (as part of biological nutrient removal) and UV treatment but not chlorine treatment. We hypothesize that the chlorine-tolerant wastewater E. coli strains possess an array of stress resistance determinants important for their overall survival in wastewater, irrespective of the specific wastewater treatment algorithms used at the WWTP, and that these strains become widely distributed throughout various municipal WWTPs. A common mechanism of resistance in these strains may center around biofilm formation coupled with an enhanced generalized stress response (RpoS), universal stress protein upregulation, and the heat shock stress response (LHR).

Only one virulence gene, *fimH*, among 28 virulence genes surveyed, was found in the wastewater E. coli isolates possessing the *uspC*-IS*30-flhDC* marker. It is not surprising that naturalized strains of E. coli in wastewater lack virulence determinants, since virulence genes are essentially host adaptation genes, i.e., genes important for adherence, colonization, invasion, intracellular survival, and/or toxin production in a particular host. Since wastewater represents a very unique environment compared to the gastrointestinal system of an animal, these virulence genes may not be necessary for survival in this matrix. It has been reported that type 1 fimbriae are critical for biofilm formation of E. coli (71, 72) and important for bacterial survival in harsh environments. The *fimH* gene encodes an adhesive protein localized at the tips of type 1 fimbriae, and it is considered a virulence gene for uropathogenic E. coli (UPEC), as it has been demonstrated to be important for colonizing the urinary tract (73). However, the absence of other UPEC-related virulence factors (74), such as uropathogenic-specific protein (*usp*), PapG adhesin (*papGI* to -*III*) of P fimbriae, P fimbriae (*papC*), S and F1C fimbriae (*sfa foc*), hemolysin (*hlyA*), iron-regulated gene A homologue adhesin (*iha*), cytotoxic necrotizing factor 1 (*cnf1*), catecholate siderophore receptor (*iroN*), and aerobactin receptor (*iutA*), in the wastewater E. coli isolates suggests that these strains are not likely UPEC. However, these strains are not the only E. coli strains present in finished wastewater, and other research has demonstrated that E. coli strains from this matrix can carry a high frequency of virulence genes (75). Therefore, the absence of most virulence genes in these strains cannot exclude the possibility that virulence genes can be acquired by these strains through transduction or horizontal gene transfer or that these strains may act as reservoirs of environmental and treatment resistance determinants (i.e., heat-resistant genomic islands) for pathogenic strains (12).

Wastewater treatment processes mimic the osmotic (dilution of feces in water), oxidative (aerobic digestion and chlorination), and UV (medium- or low-pressure UV treatment)-associated challenges commonly encountered by bacteria in the nonhost environment. Our data provide credence to the hypothesis that strains of E. coli may have evolved over time to enhance their survival across the wastewater treatment process and that these strains appear to be genetically distinct from most human and animal isolates. Further studies are required to determine (i) what mechanistic role the site-specific insertion of the IS*30* element has on phenotypic resistance in E. coli by comparing survival rates in response to osmotic, oxidative, and/or UV stress; (ii) whether regulatory anomalies exist in intergenic regions directly upstream of other universal stress protein genes (*uspA*, -*D*, -*E*, -*F*, and -*G*) or stress response elements in these strains and what role they play in enhancing treatment resistance/environmental persistence; (iii) whether these naturalized genetic strains can acquire virulence properties through transformation, transduction, or horizontal gene transfer in a wastewater matrix (or whether they can act as reservoirs for treatment resistance genes to be transmitted to pathogenic strains); and (iv) the roles of the RpoS response and the LHR in survival in a wastewater matrix.

Our data raise some potentially important public health concerns about the evolution of environmentally persistent/treatment-resistant resident populations of E. coli in wastewater. First, there is concern that treatment-resistant bacteria from upstream wastewater treatment plants may pose a risk to downstream drinking water utilities. Second, standards for wastewater treatment or reuse of wastewater warrant a careful consideration of the potential public health risks that may be associated with treatment-resistant or environmentally persistent bacteria present in effluents. It is generally assumed that the E. coli populations present in raw sewage are homogeneous in terms of their ability to survive the wastewater treatment processes. Consequently, and for simplicity from a regulatory perspective, the E. coli strains entering a wastewater treatment plant are considered biologically similar to the E. coli strains exiting the plant after treatment, and WWTP performance is determined based on bacterial reduction across the treatment train. However, our data suggest that naturalized E. coli populations exist in wastewater and that they possess environmental/treatment resistance properties that may allow them to preferentially survive the treatment process. Consequently, the influent population of E. coli is likely to be very different from the E. coli population exiting a WWTP, raising concerns about E. coli as a suitable indicator of WWTP performance. Moreover, if stress-tolerant E. coli strains acquire virulence genes or act as reservoirs for treatment resistance, then the human health risks associated with WWTP effluent exposure (i.e., reuse) may be underestimated. Based on the observation that virulence markers are common in E. coli isolates from wastewater effluents (75), it will be interesting to determine whether pathogenic strains exiting a treatment plant also possess a sophisticated stress response that enhances their survival through the treatment train.

The genetic uniqueness of the *uspC*-IS*30-flhDC*-carrying E. coli strains identified in the present study allowed for development of a sensitive endpoint PCR targeting the site-specific location of the IS*30* element in the *uspC-flhDC* intergenic region, which represents a potential novel marker of municipal wastewater contamination. At the E. coli population level, all raw wastewater samples were shown to be positive for the *uspC*-IS*30-flhDC* PCR marker, but only a proportion of the E. coli isolates present in untreated wastewater were shown to carry this marker (i.e., 5%). When raw wastewater was treated with chlorine, the prevalence of the *uspC*-IS*30-flhDC* marker in the surviving population jumped to 59%. The data suggest that a significant portion of the culturable E. coli biomass entering a WWTP is comprised of these novel, stress-resistant strains and that they persist throughout the treatment stream (i.e., even after chlorine or UV treatment). It was previously reported by Anastasi and colleagues (76) that within the raw sewage influent, only a portion of the E. coli population survives the treatment process when either UV or chlorine is used for disinfection. We are currently examining whether these strains differentially survive across the wastewater treatment process *in situ*.

Our description of a novel PCR assay specifically targeting a chlorine-tolerant wastewater E. coli subpopulation provides an interesting opportunity to adapt the assay for tracking of sources of human municipal wastewater pollution in water samples routinely submitted for public health purposes. In our study, 1-ml aliquots of Colilert test-positive E. coli samples were centrifuged, lysed by boiling, and recentrifuged, and the supernatants were analyzed for the presence of the *uspC*-IS*30-flhDC* marker by PCR. All raw wastewater samples were positive, whereas 94% of secondarily treated wastewater samples and 75% of E. coli culture-positive UV-treated samples were also positive for the *uspC*-IS*30-flhDC* marker. Conversely, only 2/71 and 3/57 surface water and groundwater samples, respectively, with culturable E. coli were positive for the *uspC*-IS*30-flhDC* marker. In cases where the *uspC*-IS*30-flhDC* marker was observed in surface water, it is possible that human wastewater may have affected the sources, as collection sites were known to be downstream of wastewater treatment plants within the watershed. In the case of E. coli-contaminated groundwater samples, it is possible that septic field discharges may have affected groundwater sources of these samples. The advantage of using the *uspC*-IS*30-flhDC* marker for evaluating whether a water source is affected by human municipal wastewater (over and above other source-tracking tools, such as Bacteroides) is that it can be applied immediately to routine water samples already positive for E. coli (i.e., Colilert test-positive samples). This alleviates the need for duplicate processing of samples and focuses the testing on only those samples positive for E. coli.

Although the evidence suggests that naturalized strains of E. coli may exist in wastewater, we cannot absolutely rule out the possibility that a fecal strain of extremely low abundance/prevalence—a strain so low in abundance/prevalence that it is currently not represented in GenBank or any NCBI genome database—may be the source of these strains in wastewater. Consequently, once deposited in the environment (i.e., sewage), such strains may then become dominant as others die off during treatment. However, naturalized strains of E. coli have been described for sand, sediment, and water (5–9), and as such, it may be presumptuous to assume that all E. coli strains observed in wastewater are of fecal origin, creating some authenticity to the emerging yet controversial concept that naturalized strains of E. coli may truly exist in wastewater and other nonhost environments.

### Conclusions.

In conclusion, the findings described herein suggest that some E. coli strains may have adapted to survive and grow within a wastewater matrix and that these populations comprise a significant proportion of the E. coli biomass entering a WWTP. The data also suggest that the PCR methods developed in this study may be useful for detection of an E. coli-based biomarker of wastewater contamination in the environment. The findings consequently raise some important considerations about the utility of E. coli as an indicator of wastewater treatment performance and influence our understanding of adaptation and evolution in this bacterial species.
